# Case report: Retroperitoneal biliary fluid collections secondary to common bile duct rupture - an unusual complication of choledocholithiasis in a child

**DOI:** 10.4103/0971-3026.41835

**Published:** 2008-08

**Authors:** Rajul Rastogi, Vaibhav Rastogi

**Affiliations:** Yash Diagnostic Center, Yash Hospital and Research Center, Civil Lines, Kanth Road, Moradabad, UP - 244 001, India

**Keywords:** Fluid collection, perforation, retroperitoneal

## Abstract

Rupture of the common bile duct (CBD) in a child secondary to choledocholithiasis is a rare event. In this article, the authors describe a child who presented with an acute abdomen due to CBD rupture, with subsequent acute retroperitoneal fluid collections, all diagnosed preoperatively on CT scan. The aim of this article is to show the pathways that such collections can take in the retroperitoneum.

Acute retroperitoneal biliary fluid collections are rare.[1] Common bile duct (CBD) perforation is one of the rare causes for it.[2] The majority of CBD ruptures have been described during infancy, presenting with abdominal distension, jaundice, peritonitis, and biliary ascites, due to perforation at the junction of the cystic duct with the CBD.[3] The authors report a child with acute biliary collections secondary to CBD perforation that occurred as a complication of choledocholithiasis; the case was preoperatively diagnosed on CT scan.

## Case History

A 10-year-old boy, presenting with acute abdomen of 2-3 days duration and signs of shock underwent a CT scan of the abdomen. Clinical history revealed abdominal pain, distension and mild jaundice. A history of trauma was ruled out. Laboratory examination revealed mildly increased total and direct bilirubin levels and raised serum alkaline phosphatase.

A plain, supine radiograph of the abdomen was unremarkable. USG of the abdomen showed a retroperitoneal collection and since the pancreas was only partly visualized, a diagnosis of acute pancreatitis was suggested.

**Figure 1:**
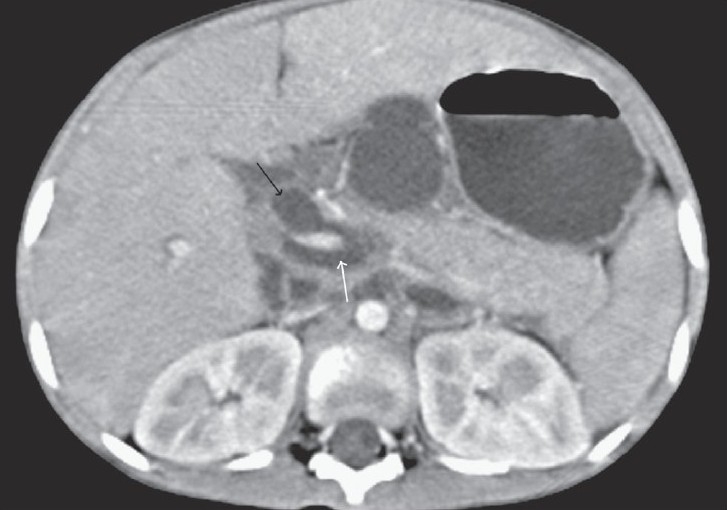
Transaxial contrast-enhanced CT scan shows a dilated suprapancreatic CBD (black arrow) communicating with a retroperitoneal collection (white arrow) that is surrounding the portal venous confluence

**Figure 2:**
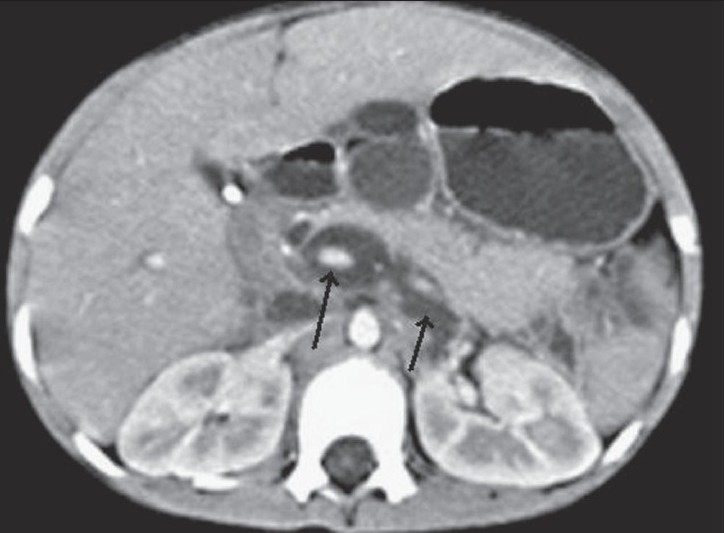
Transaxial contrast-enhanced CT scan shows a dilated intrapancreatic CBD and bile collection around the portal vein and splenic vein behind the pancreas, reaching up to the anterior left pararenal space (black arrows)

**Figure 3:**
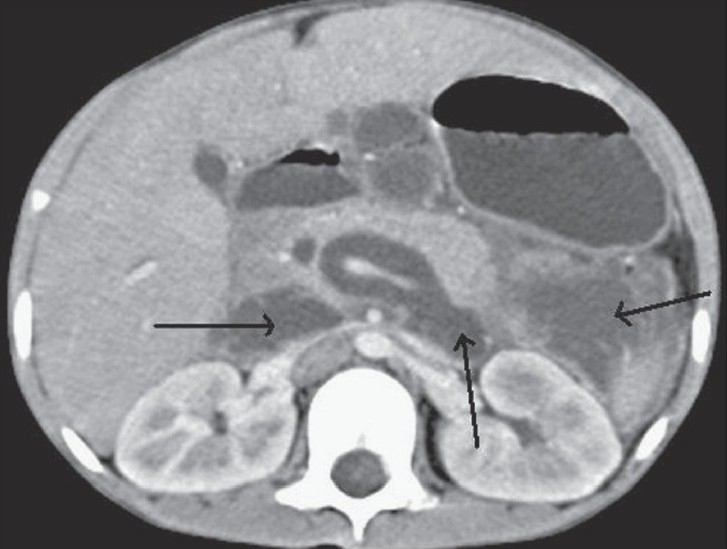
Transaxial contrast-enhanced CT scan shows a dilated intrapancreatic CBD, and a fluid collection in front of the great vessels and in the anterior left pararenal spaces (black arrows) with a pseudowall on the left

**Figure 4:**
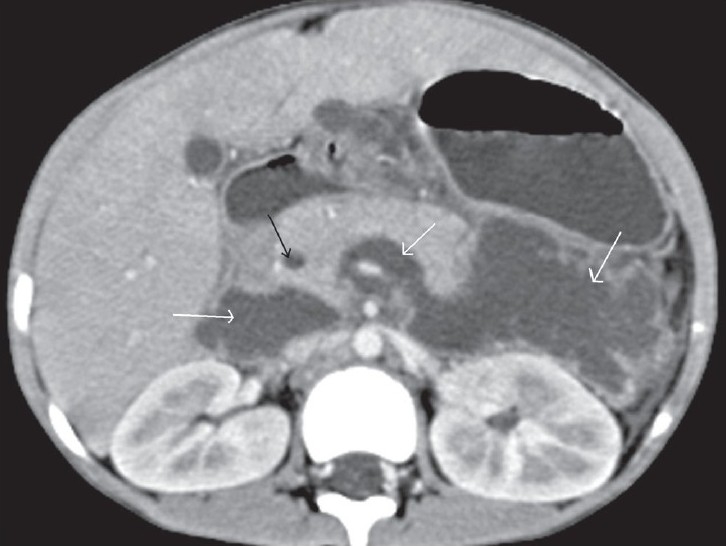
Transaxial contrast-enhanced CT scan shows a dilated intrapancreatic CBD (black arrow) and a large retroperitoneal collection on the left side of the abdomen, crossing to the right side and surrounding the superior mesenteric vein (white arrows)

**Figure 5:**
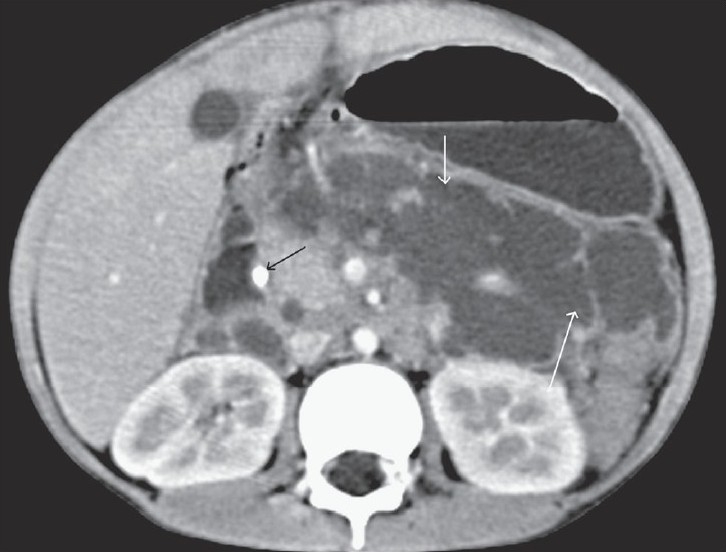
Transaxial contrast-enhanced CT scan shows a large, loculated retroperitoneal collection displacing the stomach anterosuperiorly (white arrows) with a pseudowall and septae and a calculus in the distal part of CBD (black arrow)

**Figure 6:**
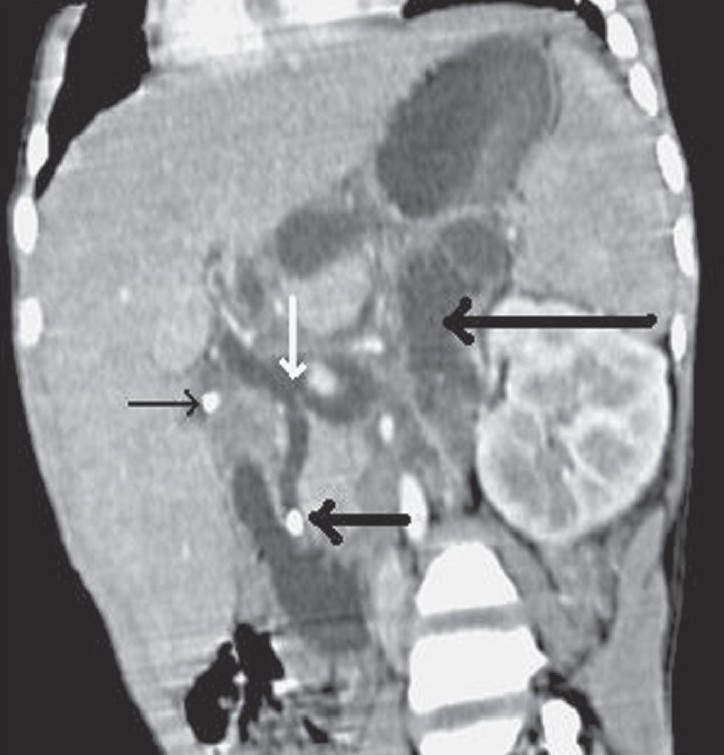
Oblique coronal MPR contrast-enhanced CT scan shows a calculus in the distal part of the CBD (thick black arrow), with proximal dilatation and a calculus in the neck of gallbladder (thin black arrow). In addition there is frank communication (white arrow) between the extrahepatic suprapancreatic CBD and retroperitoneal collections, lying between the left kidney and the pancreas and pushing the stomach superiorly (large black arrow)

CT scan of the abdomen revealed a large, loculated, retroperitoneal collection located in front of the great vessels, predominantly to the left of the midline and extending from above the level of the pancreas to the level of the lower pole of the left kidney; there was obvious communication with the extrahepatic, suprapancreatic portion of the CBD. Calculi were seen in the neck of gallbladder and in the distal part of the CBD and there was proximal biliary duct dilatation [Figures 1–6]. Minimal dilatation of the intrahepatic biliary radicles and fluid in the porta hepatis were also noted. The collection was seen surrounding the portal vein and the distal part of the splenic and superior mesenteric veins, with no intraluminal filling defects. The pancreas and rest of the abdomen were unremarkable. No evidence of ascites was noted.

Based on the clinicoradiologic findings, a provisional diagnosis of an acute retroperitoneal fluid collection, probably biliary in origin and secondary to CBD perforation, was suggested. CBD perforation was presumed to be due to increased intraluminal pressure secondary to weakening of the CBD wall due to the choledocholithiasis.

The patient was immediately operated upon. A loculated, retroperitoneal, bile-stained collection of approximately 500 ml was seen; there was communication with the dilated CBD. CBD palpation confirmed the presence of an intraductal calculus, which was removed. The collection was drained, the CBD was flushed, and a T-tube was inserted to allow for healing of the perforation. Cholecystectomy was also performed. The immediate postoperative period was uneventful. Postoperative T-tube cholangiogram and USG abdomen performed on the fourteenth postoperative day revealed no extravasation of contrast and a small retroperitoneal collection. The T-tube was removed when the cholangiogram and USG abdomen were unremarkable at the end of 4 weeks. Follow-up over the next 6 months was uneventful.

## Discussion

Perforation of the CBD usually presents in infancy,[4] mostly with peritonitis with or without biliary ascites.[4,5] Most of the cases reported are secondary to trauma to the biliary tract or a choledochal cyst. Sometimes, there may be a loculated collection which may be mistaken for a bowel duplication cyst or a choledochal cyst.[6,7] A retroperitoneal location of the biliary collection or a chronic biloma is a rarely reported complication of CBD perforation.[2,8–10] Very few cases of CBD rupture with retroperitoneal biliary collections have been reported previously and have usually been due to iatrogenic injury, postoperative biliary stricture, or choledocholithiasis.[1,11] Unlike our case, the majority of these cases have been reported in adults.

The potential space of the retroperitoneum can be divided into anterior and posterior compartments. The esophagus, duodenum, pancreas, lower two-thirds of the CBD, portal and splenic veins, appendix, ascending and descending colon, and the rectosigmoid junction are part of the anterior compartment, while the kidneys, ureters, lymph nodes, great vessels, and gonadal vessels are included in the posterior compartment.[11]

Perforation of the upper one-third of the CBD, especially at the junction of the CBD with the cystic duct (a site of congenital weakness), results in a lesser sac collection since the gallbladder, cystic duct, and upper one-third of the CBD are intraperitoneal. Perforation of the lower CBD results in a retroperitoneal collection, as was seen in our case, since this part of the CBD lies in the anterior compartment of the retroperitoneum. The anterior compartment is the potential space between the anterior renal fascia and the posterior peritoneum and hence is often referred to as the anterior pararenal space. This space communicates with the rest of the retroperitoneum through interfascial planes which are the embryologic lines of fusion of the various mesenteries. Hence, a collection in this space can spread superiorly behind the stomach adjacent to the diaphragmatic crura, laterally along the lateroconal fascia, medially across the midline into the contralateral pararenal space, posteriorly along the posterior renal fascia, anteriorly to the anterior abdominal wall through the transversalis fascia, and inferiorly along the anterolateral aspect of the psoas muscle into the pelvis, sometimes reaching the thigh or even into the scrotum in males.[1,12] Sometimes, the fluid collection may get infected, resulting in abscess formation.[11] Additional complications include thrombosis of the adjacent vasculature with resultant colonic wall necrosis.[11]

The condition remains a diagnostic dilemma in the absence of appropriate imaging studies, which may result in delayed surgical intervention and a poor prognosis.

On imaging, an acute retroperitoneal biliary fluid collection is seen as a loculated, simple or complex collection. The clue to its biliary origin is its location around the CBD or within the lesser sac or a demonstrable communication between the collection and the biliary tree. Before the advent of CT, such cases were diagnosed preoperatively only by hepatobiliary scintigraphy scans or peroperatively by cholangiography.

Early surgical management is required to treat CBD perforations and biliary collections.[13] In cases, where the preoperative or peroperative diagnosis of the site of perforation is indeterminate, external drainage is appropriate.

To summarize, perforations of the lower CBD may result in retroperitoneal biliary collections, which may track further away from the site of perforation. It is important to recognize this association.
